# P-2311. Logical Versus Absolute Lymphocyte Count-guided Preemptive Therapy for CMV Prevention in Solid Organ Transplant Recipients: A Randomized Controlled Trial

**DOI:** 10.1093/ofid/ofae631.2463

**Published:** 2025-01-29

**Authors:** Piyangkul Lorcharassriwong, Sarinya Boongird, Surasak Kantachuvesiri, Teerapat Yingchoncharoen, Napan Sutharattanapong, Jackrapong Bruminhent

**Affiliations:** Faculty of Medicine Ramathibodi Hospital, Mahidol University, Muaengsamutprakarn, Samut Prakan, Thailand; Faculty of Medicine Ramathibodi Hospital, Mahidol University, Muaengsamutprakarn, Samut Prakan, Thailand; Faculty of Medicine Ramathibody Hospital, Mahidol University, Bangkok, Krung Thep, Thailand; Faculty of Medicine Ramathibodi Hospital, Mahidol University, Muaengsamutprakarn, Samut Prakan, Thailand; Faculty of Medicine Ramathibodi Hospital, Mahidol University, Muaengsamutprakarn, Samut Prakan, Thailand; Faculty of Medicine Ramathibodi Hospital, Mahidol University, Muaengsamutprakarn, Samut Prakan, Thailand

## Abstract

**Background:**

A preemptive approach using plasma CMV DNA load monitoring is recommended among CMV-seropositive (R+) solid organ transplant recipients (SOTr). Lately, absolute lymphocyte count (ALC) has been proposed as a surrogate marker to predict CMV infection in SOTr. We hypothesized that a preemptive approach guided by immune assessment, using low ALC, could effectively prevent CMV infection, akin to the proposed routine preemptive therapy. We aimed to assess the efficacy of logical preemptive therapy (LOG) versus ALC-guided preemptive therapy for CMV infection in CMVR+ SOTr, along with cost-effectiveness.

**Methods:**

A 1:1 open-labeled randomized controlled trial was conducted at a single transplant center from February to November 2023. All adult CMVR+ SOTr not receiving anti-thymocyte globulin were randomly assigned to two groups: the LOG Group, where patients underwent plasma CMV DNA load testing every 4 weeks for 12 weeks after transplantation, and the ALC group, where the quantity of CMV DNA load was initiated when the ALC was < 1,000 cells/mm^3^. Patients were then followed for 6 months after SOT to compare the occurrence of CMV infection and the CMV DNA load testing-related cost.

**Results:**

A total of 98 eligible SOTr were included and assigned into two groups, 49 patients each. They all underwent kidney transplantation (KT) with a mean+SD age of 46+11 years old; 66.3% were male. Demographic data between the two groups were comparable. Twenty-five (25.5%) participants developed CMV infection within 6 months after KT. CMV infection was comparably observed in 13 (26.5%) participants in the LOG group vs. 12 (24.5%) participants in the ALC group, p=0.82. Of those, there were no significant differences in terms of asymptomatic CMV infection, CMV disease, and clinically significant CMV infection (p=NS, all). The total cost of plasma CMV DNA load testing (68 USD per test) was significantly lower in the ALC group compared to the LOG group (2,320 vs.10,014 USD, p< 0.01), respectively.

**Conclusion:**

Immune-guided preemptive therapy directed by low ALC appears to be effective in preventing early CMV infection after SOT. A simple and universally available ALC test could potentially be used as a personalized and cost-effective marker to initiate preemptive therapy in moderate-risk CMVR+ SOTr. TCTR20220720007.

**Disclosures:**

All Authors: No reported disclosures

